# Secreted Protein Acidic and Rich in Cysteine as a Molecular Physiological and Pathological Biomarker

**DOI:** 10.3390/biom11111689

**Published:** 2021-11-13

**Authors:** Abdelaziz Ghanemi, Mayumi Yoshioka, Jonny St-Amand

**Affiliations:** 1 Functional Genomics Laboratory, Endocrinology and Nephrology Axis, CHU de Québec-Université Laval Research Center, Québec, QC G1V 4G2, Canada; Abdelaziz.Ghanemi@crchudequebec.ulaval.ca (A.G.); mayumi.yoshioka@crchudequebec.ulaval.ca (M.Y.); 2 Department of Molecular Medicine, Faculty of Medicine, Laval University, Québec, QC G1V 0A6, Canada

**Keywords:** secreted protein acidic and rich in cysteine, expression, physiology, pathology, biomarker

## Abstract

Secreted protein acidic and rich in cysteine (SPARC) is expressed in diverse tissues and plays roles in various biological functions and processes. Increased serum levels of SPARC or its gene overexpression have been reported following numerous physiological and pathological changes including injuries, exercise, regeneration, obesity, cancer, and inflammation. Such expression pattern interrelation between these biological changes and the SPARC expression/secretion points to it as a biomarker. This property could lead to a variety of potential applications ranging from mechanistic studies and animal model validation to the clinical and therapeutic evaluation of both disease prognosis and pharmacological agents.

Secreted protein acidic and rich in cysteine (SPARC), also called BM-40 and osteonectine, is a non-collagenous [1] and collagen-binding [2], plays a non-structural role in ECM/bone [3], and has three structural domains with active glycoproteins [4] that was initially reported in bones under another name, osteonectine [1]. Additionally, studies have highlighted its implications in numerous physiological and pathological contexts at different biological levels, including in injuries and wound healing [5,6,7,8], exercise and exercise-induced muscle changes [9,10,11], glucose homeostasis and insulin secretion [12,13], metabolism and energy balance [14,15], regeneration [16], inflammation [17,18,19], cancer [20,21,22,23,24,25], obesity and diabetes [26], fibrillar collagen assembly and extracellular matrix maintenance and remodelling [17,27,28], lipid metabolism [29], immunity [30], myocardial repair and fibrosis [2], and vascular biology [31].

Importantly, SPARC protein and gene expression or its serum level changes are involved in an increase during a variety of situations. For injuries in the adult rat cerebral cortex, cortical brain injury leads to an increased expression of *Sparc* mRNA in the blood vessels [32]. While serum SPARC increases with obesity [33], its levels are reduced following bariatric surgery for weight loss [34]. In addition, the fat mass also correlates with the human adipose tissue SPARC expression [35], and *SPARC* mRNA expression in the adipose tissue is correlated to body mass index [33], which points to SPARC as a molecular indicator of the adiposity percentage. In oncology, many studies have shown that SPARC is overexpressed in different forms of cancer, including cervical carcinoma [36], colon cancer [37], and hepatocellular carcinoma [38]. Moreover, in patients with cervical carcinoma [36] or ampullary cancer [39], such overexpression is associated with a poor prognosis, and increased serum SPARC levels have been reported in melanoma patients as well [40]. Interestingly, the serum SPARC level has been proposed as a pancreatic cancer marker, as it has also been correlated to tumour size [41]. Serum SPARC levels also correlate with coronary artery lesion severity in type 2 diabetic patients with coronary heart disease [42], and newly diagnosed type 2 diabetes mellitus patients also have high plasma SPARC levels [43]. In addition to these illustrative examples, SPARC/*Sparc* overexpression has also been reported in inflammation [44], following exercise [45], and during skeletal muscle regeneration [46]. All of these elements highlight the molecular importance that SPARC has biologically. Regarding the expression pattern and how SPARC is circulated, we have hypothesized that its secretion would be involved in controlling or reducing the biological damage that is associated with the processes that initially lead to its increase (feedback-like mechanism). This is, for instance, illustrated by the increase of SPARC during both obesity and cancer as well as the SPARC properties to inhibit both adipogenesis and tumor development [14,21]. Furthermore, SPARC has been found both extracellularly and intracellularly [47] in addition to its presence in the blood, a distribution and secretion pattern that support classifying it as a biomarker.

The objective of this piece of writing is to introduce the concept of measuring SPARC protein or gene expression/level in selected biological samples as a biomarker that has potential applications in the diverse fields of biomedical research as well as in clinical practice. Indeed, since SPARC/*Sparc* expression/secretion changes with various diseases and physiological status, measuring the expression levels of SPARC (or its genes, *SPARC*/*Sparc*) or SPARC serum levels could allow the determination of how severely the disease has advanced, how efficient the treatment is, or how the pathogenesis evolves. The biological significance of such status-dependent expression patterns would lead to numerous potential biomedical and clinical applications (Figure 1). For instance, whereas high SPARC levels would reflect a disease evolution or a poor prognosis, decreased SPARC levels would be considered as an indicator of positive disease evolution or a reduced severity. The same logic applies to therapeutic evaluation in which reduced SPARC expression/level could indicate treatment efficacy. It seems acceptable to assume that the precision of such an evaluation would be higher in the tissues that express the most SPARC compared to those that express it less. Furthermore, changes during pathological phases could allow SPARC expression to be followed throughout different disease stages as a marker and thus to build a reference library for diagnosis and prognosis evaluation based on SPARC levels. Similarly, it can also be used to evaluate disease treatments since it may change with diseases improvement. Therefore, we suggest the use of SPARC as a biomolecular evaluation tool either during disease progress, treatment, or during studies aiming to evaluate disease pathogenesis. Pathogenesis exploration, mechanism studies, and animal model validations represent other applications that can be achieved through the expression of SPARC within pathway models and as a validation criterion for animal model building.

Importantly, with this expression specificity of SPARC within different pathological contexts, the potential implications of SPARC in pathogenesis are worthy of further exploration in order to identify new therapeutic targets, drugs, or adjuvants for metabolic disorders, inflammation, or cancer, especially because SPARC has been shown to play roles related to the cytotoxic effect of sorafenib against hepatocellular carcinoma cells [48]. To conclude, the interindividual differences in terms of SPARC expression in pathophysiological and therapeutic contexts can contribute to the optimization of a precision medicine supported by advanced methods in screening and sequencing. These perspectives are relevant to various applications ranging from biomolecular medical research to clinical applications. There are some challenges that have yet to be overcome. The first challenge would be the detection method and whether to use the protein level or the mRNA level as a marker. This would mainly depend on the available biological samples (sampling would depend on the patient’s physiopathological status) as well as the laboratory equipment/budget. If more than one type of sample is available, then the choice requires further studies in order to first evaluate whether the protein level and mRNA level are equivalently accurate to build standard measurement methods. Overall, we still need more in-depth studies and comparative measures to determine SPARC/*Sparc* measuring protocols for each physiological or pathological condition in order to determine the more convenient methods for use in a hospital laboratory.

Herein, SPARC/*Sparc* illustrates how identifying biomolecules and elucidating their related expression patterns based on pathophysiological variables could lead to the identification novel, yet specific, biomarkers that could be used as parameters for diagnosis, prognosis, therapeutic tests, and clinical evaluation.

## Figures and Tables

**Figure 1:**
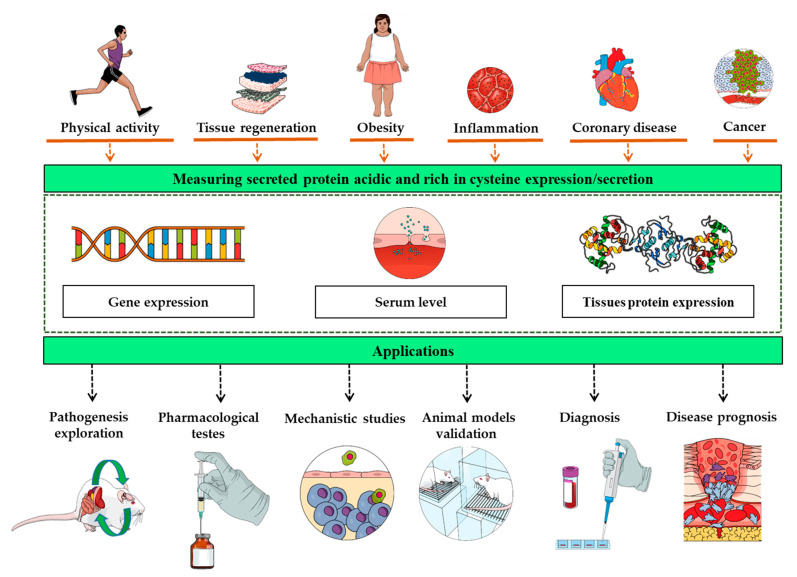
The overexpression of the secreted protein acidic and rich in cysteine gene, protein, or its increased blood concentration follow numerous physiological and pathological changes including exercise, obesity, cancer, injuries, and inflammation. Such interrelation between these biological changes and the secreted protein acidic and rich in cysteine expression/secretion points to it as a biomarker with a variety of potential applications, ranging from mechanistic studies to the clinical and therapeutic evaluation of both disease prognosis and pharmacological agents.

## Data Availability

Not applicable.
